# Exploration of Lipid Metabolism in Gastric Cancer: A Novel Prognostic Genes Expression Profile

**DOI:** 10.3389/fonc.2021.712746

**Published:** 2021-09-08

**Authors:** Zhen Xiong, Yao Lin, Yan Yu, Xianghui Zhou, Jun Fan, Colin J. Rog, Kailin Cai, Zheng Wang, Zhijie Chang, Guobin Wang, Kaixiong Tao, Ming Cai

**Affiliations:** ^1^ Department of Gastrointestinal Surgery, Union Hospital, Tongji Medical College, Huazhong University of Science and Technology, Wuhan, China; ^2^ Department of Breast and Thyroid Surgery, The Central Hospital of Wuhan, Tongji Medical College, Huazhong University of Science and Technology, Wuhan, China; ^3^ Department of Hematology, Union Hospital, Tongji Medical College, Huazhong University of Science and Technology, Wuhan, China; ^4^ Department of Pathology, Union Hospital, Tongji Medical College, Huazhong University of Science and Technology, Wuhan, China; ^5^ Department of General Surgery, Swedish Medical Center, Seattle, WA, United States; ^6^ State Key Laboratory of Membrane Biology, School of Medicine, Tsinghua University, Beijing, China

**Keywords:** lipid metabolism, gastric cancer, prognosis, genes profile, validation

## Abstract

**Background:**

Alterations in lipid metabolism are increasingly being recognized. However, the application of lipid metabolism in the prognosis of gastric cancer (GC) has not yet been explored.

**Methods:**

A total of 204 lipid metabolism relative genes were analyzed in the GC cohort from The Cancer Genome Atlas (TCGA), and four independent cohorts from Gene Expression Omnibus (GEO) and one cohort from Wuhan Union Hospital were applied for external validation. Differential expression and enrichment analyses were performed between GC and normal tissue. The LASSO-Cox proportional hazard regression model was applied to select prognostic genes and to construct a gene expression profile.

**Results:**

Our research indicated that higher expression level of AKR1B1, PLD1, and UGT8 were correlated with worse prognosis of GC patients, while AGPAT3 was correlated with better prognosis. Furthermore, we developed a gene profile composed of AGPAT3, AKR1B1, PLD1, and UGT8 suggested three groups with a significant difference in overall survival (OS). The profile was successfully validated in an independent cohort and performed well in the immunohistochemical cohort. Furthermore, we found that ether lipid metabolism, glycerophospholipid metabolism, and glycerolipid metabolism were upregulated, and fatty acid β-oxidation and other lipid peroxidation processes were reduced in GC.

**Conclusion:**

Collectively, we found lipid metabolism is reliable and clinically applicable in predicting the prognosis of GC based on a novel gene profile.

## Introduction

Gastric cancer (GC) is a leading cause of cancer-related death, especially in Eastern Asia (1–3). Despite significant improvements in the survival of patients with GC over the past several decades, most of GC patients were already in an advanced stage at the time of diagnosis with poor prognosis (4). At present, prognostic indicators of GC mainly rely on TNM staging system. Nevertheless, several studies have demonstrated significant differences in clinical outcomes among patients with similar TNM stages receiving treatment regimens, suggesting that the TNM staging system may not completely predict the prognosis of patients with GC when used alone (5, 6). Newly available molecular data may help identify more specific biomarkers that help to categorize patients with similar TNM staging but differing prognoses.

Lipids, also known as fats, are required for energy storage, membrane proliferation, and the generation of signaling molecules. Alterations in lipid metabolism in cancer cells have received increasing attention and recognition. Accumulating evidence has demonstrated that cancer cells commonly have characteristic changes in lipid metabolism (7–9). Lipogenesis, for example, is strongly upregulated to satisfy the demands of increased membrane biogenesis in malignant tumors (9), and lipid uptake and storage are also disproportionately elevated (10, 11). Targeting these pathway-regulating lipid metabolisms has become a novel anticancer strategy. Although some single biomarkers of lipid metabolism have been reported to be associated with tumor prognosis, none has been consistently validated.

In this research, we demonstrate differences in lipid metabolism pathways between GC and normal gastric tissue and further demonstrate an associated correlation with the prognosis of GC. Additionally, a set of lipid metabolism genes associated with prognosis was found by correlation analysis of several gastric cancer cohorts. Finally, we explored a prognostic signature based on these lipid metabolism genes which highlights the potential to improve GC precision therapy.

## Materials and Methods

### Patients and Cohorts

**Figure 1:**
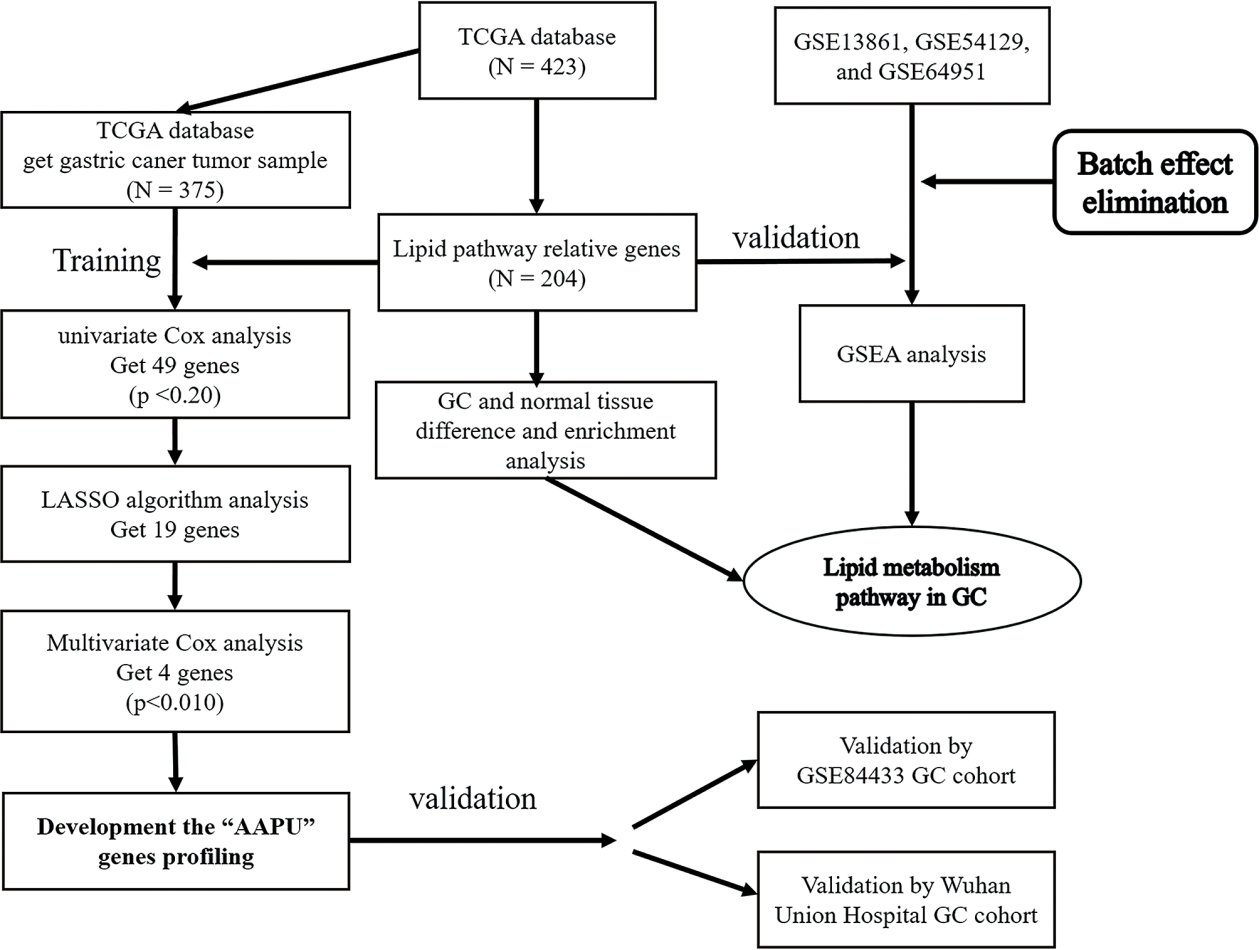
Study design.

One thousand one hundred twenty-nine GC samples from six independent datasets were analyzed in this research, including GSE13861 (12), GSE54129 (13), GSE64951 (14), and GSE84433 (15) from the Gene Expression Omnibus (GEO) datasets (http://www.ncbi.nlm.nih.gov/gds, RRID: SCR_005012), one dataset from The Cancer Genome Atlas (TCGA), and one cohort from Wuhan Union Hospital. To maintain consistency, all of the datasets from the GEO were processed using the same chip platform (Affymetrix Human Genome U133 Plus 2.0 Array, Santa Clara, CA, USA) which has been extensively used for transcriptome analysis and has numerous advantages. This chip platform has high accuracy and reproducibility for each transcript. The training dataset contained 375 GC samples accessed from TCGA (level III gene expression data, combining published and provisional GC samples (https://genome-cancer.ucsc.edu/). Similarly, the validation dataset (GEO cohort) was composed of an adequate number of GC samples (GSE84433). Furthermore, we applied another validation dataset (WH cohort) from Wuhan Union Hospital that contained 81 fresh frozen primary GC samples consecutively collected at Wuhan Union Hospital from January 2012 to January 2019 (**Figure 1**).

The study was approved by the ethics committee of Union Hospital, Tongji Medical College, Huazhong University of Science and Technology, Wuhan, China. Written informed consent was obtained from patients enrolled in the study. The study conformed to the provisions of the Helsinki Declaration. None of the patients had received radiotherapy or chemotherapy prior to surgery.

### Extraction of Genes in Lipid Metabolism

Through searching lipid metabolism pathways in the Kyoto Encyclopedia of Genes and Genomes (KEGG) database (https://www.kegg.jp/), genes related to lipid metabolism were extracted from hsa00061, hsa00062, hsa00071, and other KEGG pathways. After eliminating repetitive, nonsense, and polysemous gene annotations, we constructed a lipid metabolism relative gene database.

### Differentially Expressed Gene Analysis Between Tumor and Normal Samples

Initially, differential tests were performed on GC and adjacent normal mucosa tissues. Differentially expressed genes (DEGs) between GC and adjacent normal mucosa tissue samples from TCGA dataset were screened with the thresholds of *Q*-value (adjusted *p*-value between two groups) <0.05 and |Log2 fold change (FC)| >1.50 using the “limma” package in R (16). The DEGs with |Log2 FC| >1.50 was identified as the cancer-specific gene.

### Construction of Multigene Profile Based on LASSO-Cox Algorithm

The “glmnet” package in R was utilized to perform the Cox regression analysis with the least absolute shrinkage and selection operator (LASSO) algorithm (17). We applied LASSO algorithm divide genes into two subtypes that one is a positive correlative with events and one is a negative correlative with events. After that, robust markers were selected from candidate genes in two subtypes by the LASSO algorithm, in which the datasets were subsampled and the tuning parameters were determined according to the expected generalization error estimated from 10-fold cross-validation. Then, a multivariate Cox regression analysis with the stepwise method based on the Akaike information criterion (AIC) calculation was conducted to screen the independent prognostic factors in robust markers (18). The genes profile was constructed by calculating the expression values of the selected genes weighted by their corresponding coefficients in the multivariate Cox regression analysis. Cox proportional hazard analysis was utilized to obtain the *β*-score of every gene and *P <*0.01 of every *β*-score was conferred priority.


$$\text{Risk Score}=\beta_{1}X_{1}+\beta_{2}X_{2}+\beta_{3}X_{3}+\ldots +\beta_{n}X_{n}$$

The risk score was calculated for each sample, then the value of the risk score was determined to distinguish the low-, moderate-, and high-risk group by X-tile (19).

### External Validation of Multigene Profiles by GEO Analysis

Kaplan–Meier with the log-rank test was applied to show the survival difference between different groups in dataset GSE84433. Furthermore, our research performed the correlation analysis between the clinical stage of GC patients and selected the gene profile.

### Immunohistochemical Expression Analysis

Immunohistochemistry and evaluation were performed as described. Rabbit polyclonal antibodies for AGPAT3(PA5-49623), AKR1B1(PA5-82915), and UGT8 (PA5-48251) were from Invitrogen (San Diego, CA, USA). Mouse monoclonal PLD1(sc-28314) was from Santa Cruz Biotechnology (Santa Cruz, CA, USA). The expression status was defined as detectable immunoreaction in perinuclear and/or cytoplasm and was semiquantitatively estimated from 1 to 3: 1, 0%–39% positive cancer cells; 2, 40%–69% positive cancer cells; and 3, ≥70% positive cancer cells (20). The expression status of each case was assessed by two independent observers. All observers were unaware of the purpose of the study.

### Genes Enrichment and Pathway Analysis in TCGA and GSE Datasets

To explore the expression of lipid metabolism pathway, GSEA was performed using a Java GSEA desktop application that was downloaded from http://www.bro-ad.mit.edu/gsea/ (21). Three GSE dataset was analyzed with the GMT file (c2. KEGG. v8.2) gene set to obtain biological processes enriched by biomarkers in prognosis profile. A total of four files including expression datasets, gene sets, phenotype labels, and chip platforms were loaded for running GSEA according to the manufacturer’s specifications. False-discovery rate (FDR) <0.05 was identified to be significantly enriched, and the significantly enriched KEGG pathways were visualized using GSEA v4.1.0 software.

### Statistical Analysis

We assessed the correlation between lipid metabolism relative genes and clinicopathological features using independent-samples *t*-test and Chi-square test. Kaplan–Meier survival analysis and log-rank test were used to estimate the prognosis of GC patients. A Cox proportional hazards model was used to perform standard univariate and multivariate analyses. Prediction error curves were used to compare the accuracy of survival models. The Cox regression coefficients were used to construct the profile. Calibration plots were generated to explore the performance characteristics of the profile. In the calibration plot, the *x*-axis indicates predicted survival probability and the *y*-axis indicates the actual freedom from overall survival (OS) for the patients. Time-dependent receiver operating characteristic (ROC) analysis was performed to assess the predictive accuracy. All the statistical tests were performed with R software (version 4.0.1, Auckland, New Zealand) and Prism 8 software (version 8.02, Charlotte, NC, USA). Statistical significance was set at bilateral 0.05.

## Results

### Expression of Lipid Metabolism Relative Genes Between Gastric Cancer and Adjacent Normal Mucosa Samples

**Figure 2:**
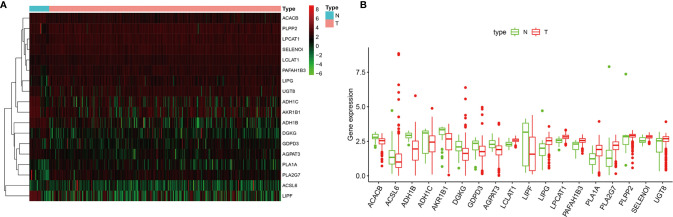
**(A)** The heatmap of differentially expressed genes (DEGs) between gastric cancer (GC) and normal tissues. **(B)** Boxplots of mRNA expression of DEGs.

A cohort containing 375 gastric cancer patients with available expression data and clinical information in the TCGA database was analyzed. A total of 204 genes relative to lipid metabolism, extracted from KEGG dataset, are shown in **Table S1**. Under the criteria that *p* < 0.05 and |Log2 FC| ≥1.50, 18 differentially expressed genes (DEGs) were screened (**Figure 2A**), including ACACB, ACSL6, ADH1B, ADH1C, AKR1B1, DGKG, GDPD3, AGPAT3, LCLAT1, LIPF, LIPG, LPCAT1, PAFAH1B3, PLA1A, PLA2G7, PLPP2, SELENOI, and UGT8. The difference in expression level of mRNAs between GC and normal tissues is shown in **Figures 2A, B**.

### Identification of Lipid-Metabolism Cancer-Specific Multigene Profile for Prognostic Prediction in Gastric Cancer

**Figure 3:**
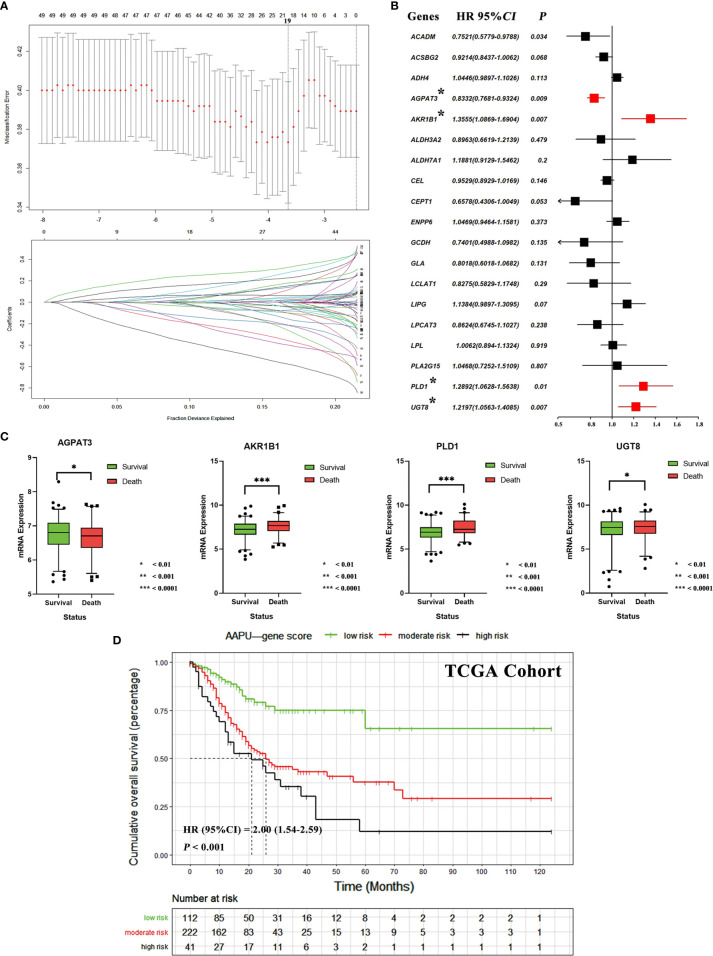
**(A)** Tuning parameter (*λ*) selection in the LASSO algorithm performed using 1,000-fold cross-validation *via* the minimum criteria. The LASSO coefficient profiles of the 19 genes. A coefficient profile plot is produced versus the log (*λ*). The binomial deviance is plotted versus log (*λ*) and the black vertical lines are plotted at the optimal *λ* based on the minimum criteria. **(B)** The forest plots of multivariate Cox analysis results of 19 genes selected by LASSO algorithm. **(C)** The mRNA expression difference of AGPAT3, AKR1B1, PLD1, and UGT8 between alive and dead GC patients. **(D)** The KM survival curves of low-, moderate- and high-risk “AAPU” genes profile in TCGA cohort.

We identified 49 lipid metabolism relative genes in GC patients by univariate Cox analysis (adjusted *p*-value <0.05) (**Table S2**). We then used a LASSO regression model to extract 19 survival relative genes (**Figure 3A**). Finally, our study developed a prognostic profile that selected four out of the 19 genes by multivariate Cox analysis (*p*-value < 0.010) identified in the training dataset (**Figure 3B**). It existed low correlations between the expression level of the four genes (**Figure S1**). Through difference analysis of survival, results indicated the expression of AKR1B1, PLD1, and UGT8 were death risk relative genes, while AGPAT3 was expressed higher in GC patients with better prognosis (**Figure 3C**).

Using the LASSO-Cox regression model, we then derived a risk score for each patient based on the individual expression levels of the four genes, namely, AAPU = −0.1826* expression level of AGPAT3 + 0.3042* expression level of AKR1B1 + 0.2540* expression level of PLD1 + 0.1986* expression level of UGT8. Using X-tile plots, patients in the training dataset were classified into low, moderate, and high AAPU group with an optimum cutoff value of 4.0 and 4.8 after AAPU was normalized. The KM survival curve analysis demonstrated that the three groups had significantly different outcomes (HR = 2.00; 95% CI, 1.54–2.59; *p* < 0.001; **Figure 3D**). Moreover, martingale residuals plots and Schoenfeld individual test plots showed balanced hazard proportionality in the AAPU (**Figure S1**).

### The AAPU Profile and TN Staging in GEO Cohort

**Figure 4:**
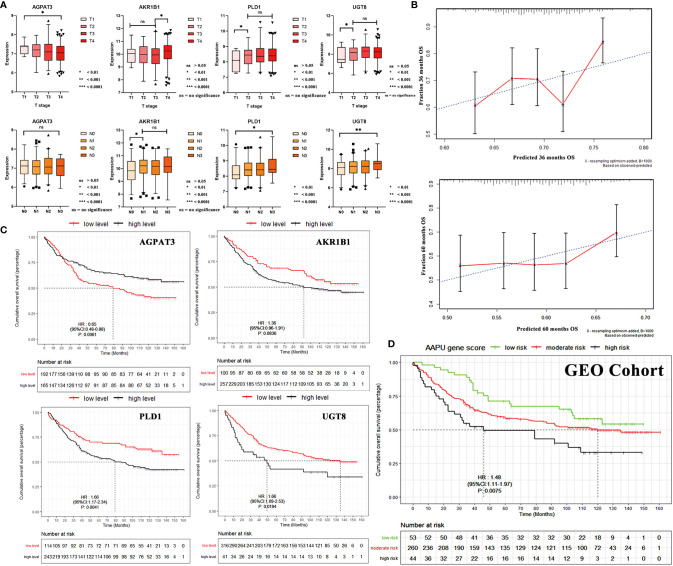
**(A)** The mRNA expression correlation between AGPAT3, AKR1B1, PLD1, and UGT8 of TN stage in the validation cohort. **(B)** Three- and 5-year calibration curves of AAPU profile. **(C)** KM survival curves of low- and high-level AGPAT3, AKR1B1, PLD1, and UGT8 in the validation cohort. **(D)** The KM survival curves of low-, moderate-, and high-risk “AAPU” genes profile in the GEO cohort.

Stratification analyses were performed in the validation cohort of patients grouped by T and N stages. With the increase in the T stage, there existed a significant increase in the mRNA expression of AKR1B1, PLD1, and UGT8. Specifically, a significant increase existed between T1–3- and T4-stage GC patients for PLD1 and UGT8. On the other hand, the expression of AGPAT3 was reduced. As for the N stage, AKR1B1, PLD1, and UGT8 were noted with higher N stage. Specifically, a significant increase existed between N0- and N1–3- stage GC patients for AKR1B1 and PLD1. There was no significant difference for AGPAT3 (**Figure 4A**).

### Validation of AAPU Profile for Predicting Survival in TCGA and GEO Cohorts

To confirm that the proposed AAPU prefiling has a similar prognostic value in different populations, the same formula was applied to the training cohort (TCGA) from the USA and validation cohort (GSE84433) from Asia. The prognostic accuracy of the AAPU profile as a continuous variable in these cohorts was also assessed using time-dependent ROC analysis. To make an internal validation using the training dataset, the C-index was 0.734 (95% CI, 0.672–0.798) for the prognostic profile, and with an AUC of 0.721, the prognostic AAPU profile showed an excellent discrimination capacity in predicting the 5-year OS. Conversely, to perform external validation using the validation dataset, the C-index was 0.674 (95% CI, 0.621–0.723) for the prognostic profile, and with an AUC of 0.652, the AAPU profile performed consistently as well. Additionally, the time-dependent ROC curve of the prognostic AAPU profile was found to be consistently more favorable in both training and validation cohorts (**Figure S2**). Furthermore, the calibration curves indicated that the prognostic AAPU profile predicted the 3- and 5-year OS of the GC patients in the validation cohort accurately (**Figure 4B**). Moreover, consistent with the findings in the training cohort, patients in the different AAPU profile groups had a significant difference in overall survival rate in the validation cohort (HR = 1.48; 95% CI, 1.11–1.97; *p* < 0.001) (**Figures 4C, D**).

### The Immunohistochemical Score of AAPU Profile and GC Patient Survival in WH Cohort

**Figure 5:**
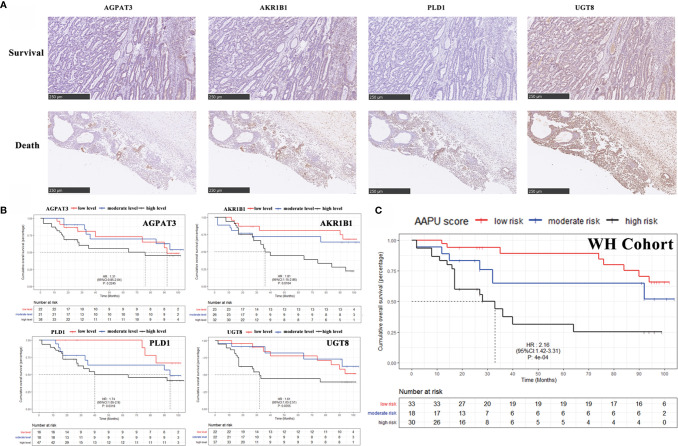
**(A)** Representative images of IHC for AGPAT3, AKR1B1, PLD1, and UGT8 in GC case with a H&E staining, ×10. **(B)** KM survival curves of low- and high-level AGPAT3, AKR1B1, PLD1, and UGT8 in Wuhan Union Hospital cohort. **(C)** The KM survival curves of low-, moderate- and high-risk “AAPU” genes profile in the WH cohort.

To validate the expression level in protein, our study detected the AGPAT3, AKR1B1, PLD1, and UGT8 expression of protein level in 81 tumor samples of GC patients (**Figure 5A**) in the WH cohort. The KM analysis showed the higher protein expression index of AKR1B1, PLD1, and UGT8 in GC patients with worse OS, but no significant relevance between the protein expression index of AGPAT3 and prognosis in GC patients (**Figure 5B**). Furthermore, there were significant differences between the early (I–II) and late (II–IV) clinical stages in the protein expression index of AKR1B1, PLD1, and UGT8 (*p* < 0.001). Moreover, the AAPU profile performed well in the immunohistochemical WH cohort (HR = 2.16; 95% CI, 1.42–3.31; *p* < 0.001) (**Figure 5C**).

### Enrichment Analysis of the Lipid Metabolism Pathway in GC

**Figure 6:**
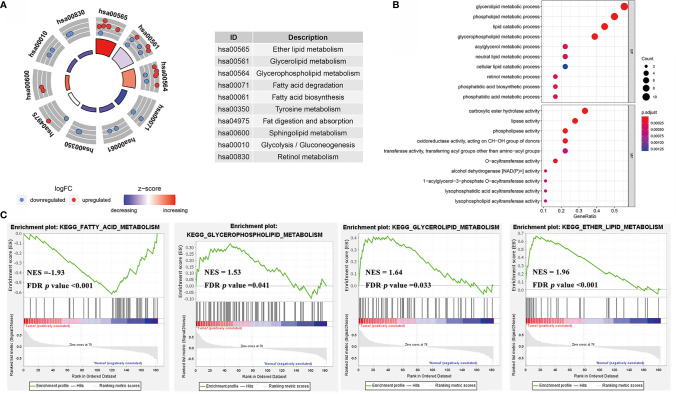
**(A)** The circle diagrams of KEGG pathway enrichment. **(B)** The bubble diagrams of GO terms enrichment. **(C)** The gene set enrichment analysis (GSEA) curves of KEGG pathways of lipid metabolism in GC rather to normal tissues.

GO and KEGG pathway enrichment analyses was performed with a threshold of *p* < 0.05 (**Figures 6A, B**) in the TCGA cohort. The results of the KEGG pathway analysis revealed that these genes were primarily enriched in ether lipid metabolism, glycerolipid metabolism, and glycerophospholipid metabolism Furthermore, glycerolipid metabolic process, phospholipid metabolic process, and lipid catabolic process were upregulated in GC compared with normal tissue. The results indicated that the significantly enriched GO terms for BP were carboxylic ester hydrolase activity, lipase activity, and phospholipase activity oxidoreductase activity were enriched.

**Figure 7:**
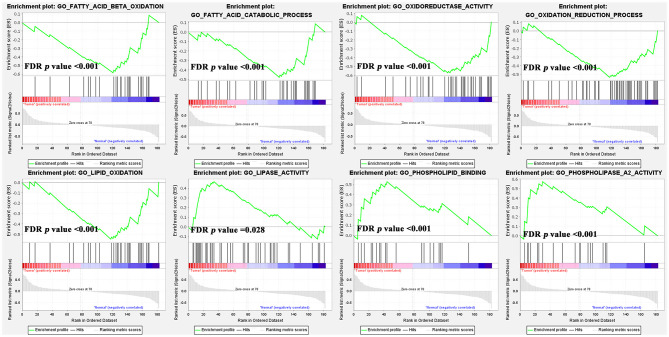
The gene set enrichment analysis (GSEA) analysis of GO terms of lipid metabolism in GC rather than normal tissues.

Furthermore, our study combined GSE13861, GSE54129, and GSE64951 with a batch effect elimination to externally validate the enrichment results (**Figure S3**). As the GSEA analysis results indicated, ether lipid metabolism, glycerophospholipid metabolism, and glycerolipid metabolism were upregulated in GC compared with normal tissue, while fatty acid metabolism was downregulated (**Figure 6C**). As for GO terms in GSEA analysis, fatty acid *β*-oxidation, oxidation-reduction process, lipid oxidation, and oxidoreductase activity were reduced (**Figure 7**).

## Discussion

Over the last several years, it has been widely reported that increased lipid uptake, storage, and lipogenesis occur in a variety of cancers and contribute to rapid tumor growth (22–25). However, although several gene expressions of lipid metabolism have demonstrated a significant role in tumor proliferation (22, 24), no studies have reported effects on patient prognosis.

In our analysis, multiple pathways of lipid metabolism differed significantly between GC and normal tissues (**Figure 2**). It is noteworthy that among these significantly different pathways, fatty acid (FA) oxidation-related pathways, such FA *β*-oxidation and FA metabolism, showed a significant downregulation in tumor tissues, while other pathways, such as phospholipids and glycerol lipids, showed opposite upregulation (**Figure 3**). Based on the “Warburg effect,” increased glucose consumption, glycolytic activity, and lactic acid accumulation are important markers of tumors (26). Currently, some studies have demonstrated that glucose concentration in GC tissues is very low, while lactic acid and other products of glycolysis are much higher than in normal tissues (27). It was also reported that lactic acid increased significantly in gastric cancer tissues, while citric acid, malic acid, and succinic acid decreased significantly, indicating that glycolytic activity increased while the tricarboxylic acid cycle decreased significantly in gastric cancer tissues (28). Taken together, these abnormal activation pathways suggest that lipid metabolism is more involved in signal transmission than energy supply in tumor biological behavior, highlighting the potential application value of lipid metabolism in predicting GC prognosis.

In this paper, we assessed those pathways and identified a set of genes that were the most significant predictors of prognosis. This profile of genes includes AGPAT3, AKR1B1, PLD1, and UGT8, all of which have previously been reported to be involved in tumor proliferation. Wu et al. reported that AKR1B1 promotes basal-like breast cancer (BLBC) progression by a positive feedback loop that activates the EMT program (29). Several pieces of evidence suggest that the expression of AKR1B1 varies greatly in different stages of colorectal cancer (CRC) (30, 31). PLD1 was reported to have an important role in sustaining cancer cell survival during metabolic stress in our previous study (32). Additionally, Cao et al. reported the inhibition of UGT8 suppresses BLBC progression (33). In this study, the set of genes identified demonstrated a strong correlation with gastric cancer prognosis, and these genes could potentially be targeted by anticancer metabolism therapies in the future. By LASSO algorithm and Cox regression, we developed a novel gene profile based on the assessment of those four genes. This prognostic gene profile categorized patients with different prognoses and showed excellent efficacy in both the training set and the validation set.

To further verify the clinical application value of this prognostic profile, we conducted a retrospective analysis of GC patients in our center and applied an immunohistochemical score to verify the efficacy of the four genes at the protein level. At the protein level, all the other three genes except AGPAT3 showed a correlation with prognosis. This is largely due to the small size of the cohort and differences in clinical characteristics, such as age and ethnicity, from the database. We also consider that protein expression is also influenced by other factors, such as posttranscriptional regulation, that we did not study. Interestingly, the assumed new prognostic profile could well classify GC patients with different prognosis, indicating this novel prognostic profile has appropriate applicability and reliability. Thus, the prognostic profile identified in our study is a good complement to the current incomplete prognostic evaluation.

## Conclusion

In conclusion, in this study, we explored a novel prognostic profile based on lipid metabolism that performed well in predicting the prognosis of GC. Although further prospective studies in bigger cohorts are needed to validate the utility of the profile, our approach provides a view of GC prognosis prediction from a lipid perspective.

## Data Availability Statement

The original contributions presented in the study are included in the article/**Supplementary Material**. Further inquiries can be directed to the corresponding authors.

## Ethics Statement

The studies involving human participants were reviewed and approved by ethics committee of Wuhan Union Hospital, Tongji Medical College, Huazhong University of Science and Technology. The ethics committee waived the requirement of written informed consent for participation.

## Author Contributions

Conceptualization: MC and KT. Methodology: YL. Software: YL. Validation: ZX, YY, and XZ. Formal analysis, ZX. Investigation, JF. Resources, CR. Data curation: KC. Writing (original draft preparation): ZX. Writing (review and editing): CR and MC. Visualization: ZW. Supervision: ZC. Project administration: GW. Funding acquisition: KT. All authors contributed to the article and approved the submitted version.

## Funding

This study was supported by grants from the National Natural Science Foundation of China (No. 81972881) and Natural Science Foundation of Hubei Province of China (WJ2017M123 and 2019CFB514).

## Conflict of Interest

The authors declare that the research was conducted in the absence of any commercial or financial relationships that could be construed as a potential conflict of interest.

## Publisher’s Note

All claims expressed in this article are solely those of the authors and do not necessarily represent those of their affiliated organizations, or those of the publisher, the editors and the reviewers. Any product that may be evaluated in this article, or claim that may be made by its manufacturer, is not guaranteed or endorsed by the publisher.

## Supplementary Material

Supplementary Figure 1
**(A)** Multivariate correlation analysis of AGPAT3, AKR1B1, PLD1, UGT8, and other demographic characteristics. **(B)** Martingale residual plots of AAPU profile in the TCGA cohort. **(C)** Schoenfeld individual test plots of AAPU profile in the TCGA cohort.

Supplementary Figure 2 Time-dependent ROC curves of the prognostic AAPU profile in both the training and validation cohorts.

Supplementary Figure 3 Plots of GSE13861, GSE54129, and GSE64951 before and after bath effect elimination.
