# Supplementary figures and images for: Exploration of Lipid Metabolism in Gastric Cancer: A Novel Prognostic Genes Expression Profile
Source: Front Oncol. 2021 Sep 8;11:712746. doi: 10.3389/fonc.2021.712746 (PMC8457048; doi:10.3389/fonc.2021.712746)

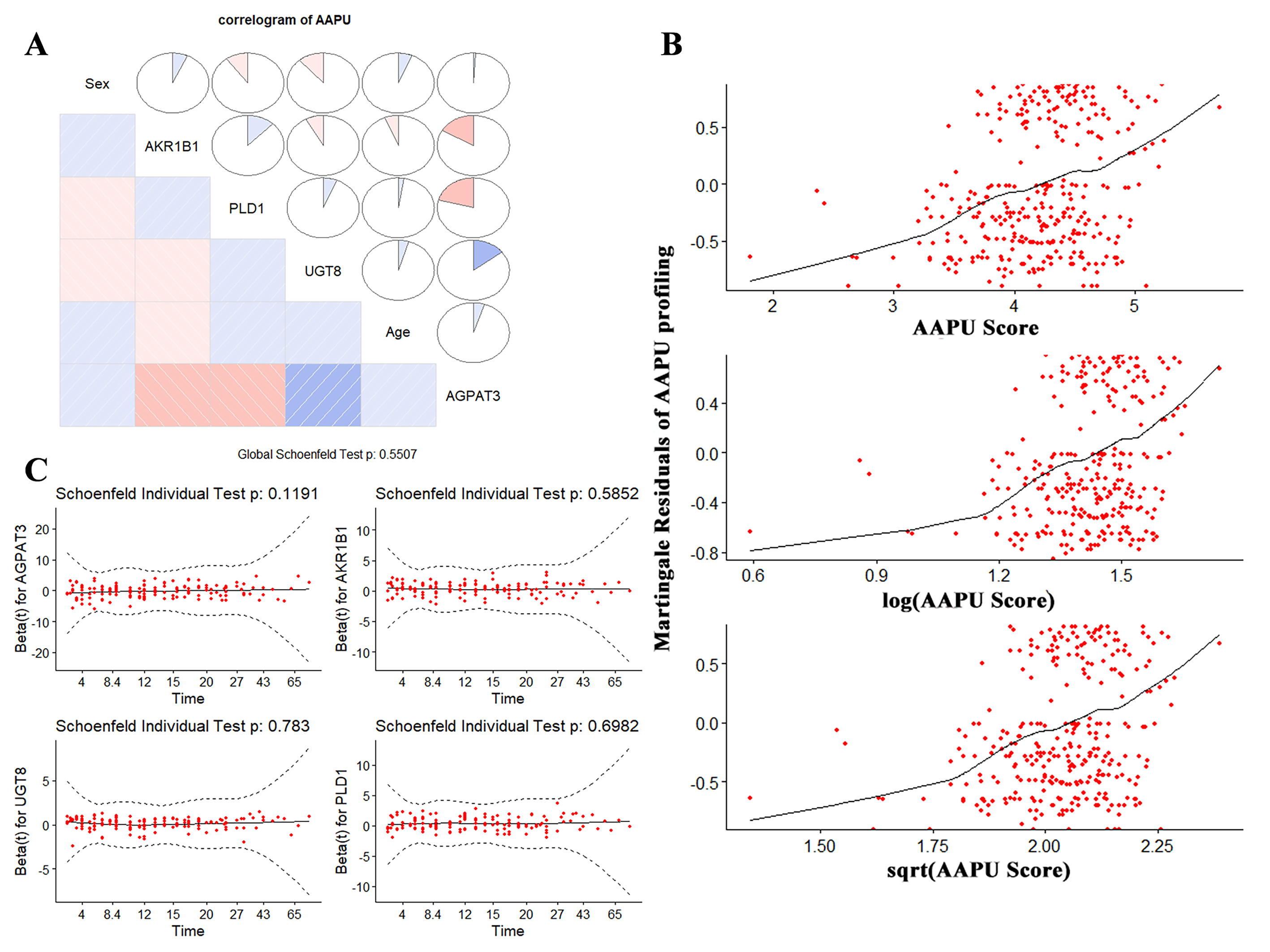

Supplement: Supplementary Figure 1 — (A) Multivariate correlation analysis of AGPAT3, AKR1B1, PLD1, UGT8, and other demographic characteristics. (B) Martingale residual plots of AAPU profile in the TCGA cohort. (C) Schoenfeld individual test plots of AAPU profile in the TCGA cohort. [file Image_1.tif]

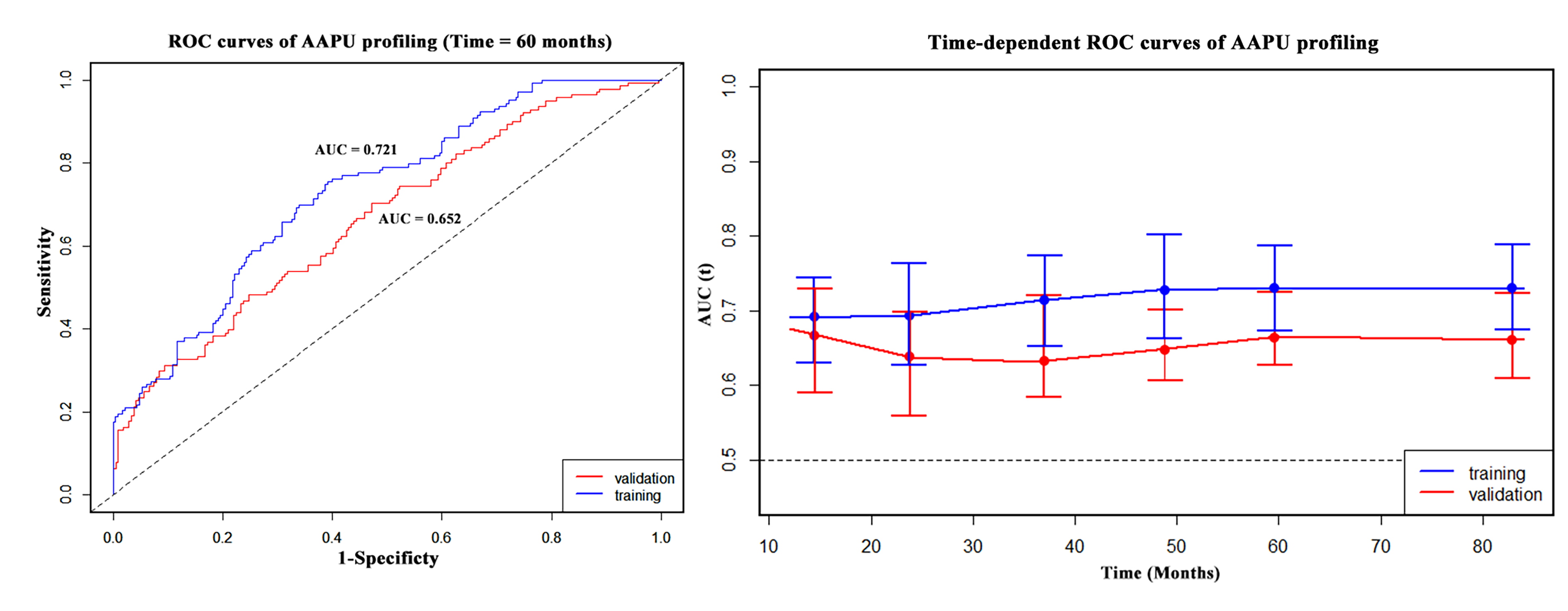

Supplement: Supplementary Figure 2 — Time-dependent ROC curves of the prognostic AAPU profile in both the training and validation cohorts. [file Image_2.tif]

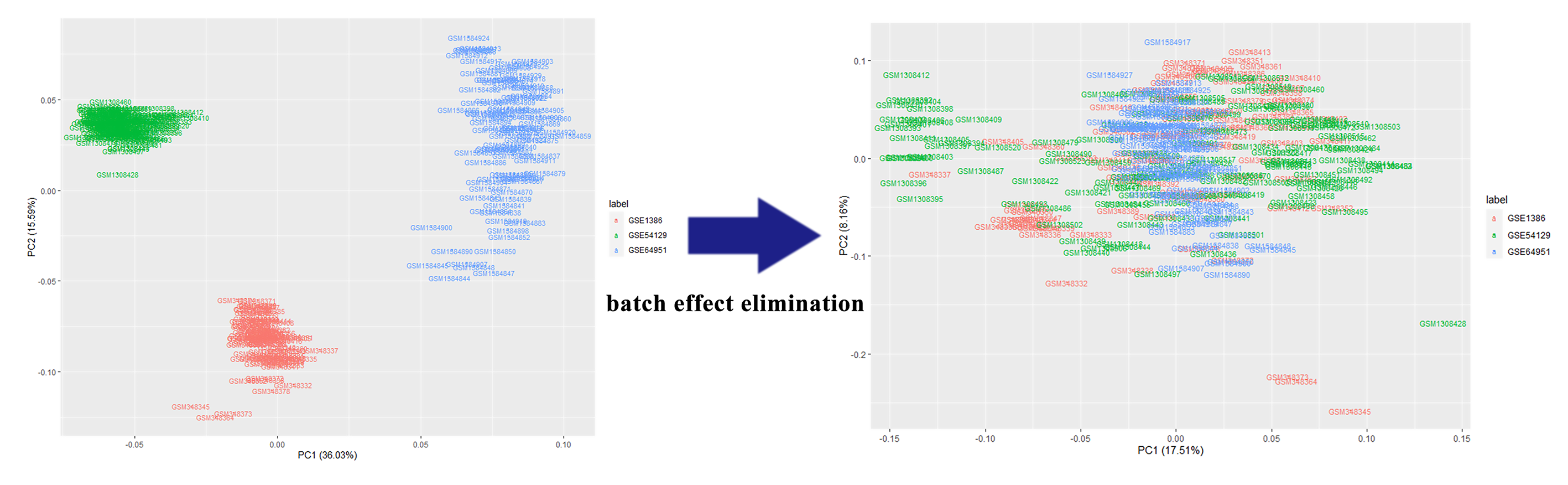

Supplement: Supplementary Figure 3 — Plots of GSE13861, GSE54129, and GSE64951 before and after bath effect elimination. [file Image_3.tif]
